# Delirium Produced by Salicylic Acid

**Published:** 1882-05

**Authors:** 


### DELIRIUM PRODUCED BY SALICYLIC ACID.

It does not seem to be generally known that salicylic acid given in large doses every two hours will occasionally produce quite active delirium. Dr. C. C. Barrows, late House Physician, Bellevue Hospital (*Medical Record,* April 29, 1882), reports eight cases of delirium, resembling very closely that of acute alcoholism, following the administration of salicylic acid in doses of twenty grains every two hours. On omitting the acid the delirium at once subsided. In the above cases the acid was given for acute rheumatism.

